# *PLoS Medicine—* A Medical Journal for the Internet Age

**DOI:** 10.1371/journal.pmed.0010031

**Published:** 2004-10-19

**Authors:** Michael B Eisen, Patrick O Brown, Harold E Varmus

## Abstract

A message from the founders of the Public Library of Science

The Internet is awash with medical information. Eight hundred million people have direct access to the Internet [1], and in the United States over 60% have searched for health or medical information on the Web [2]. Go to any search engine and type in the name of a disease or drug, and you will be directed to hundreds of sites, ranging from the sound and useful to the quackish and dangerous. Google “medical” and you get 85 million pages, “drug,” 40 million, and “health,” 230 million.

But something is conspicuously missing. The most reliable medical information on the Internet—the contents of peer-reviewed medical journals—is hidden from the public and most of the world's physicians. Although most medical journals are available online, their publishers limit access to those who choose, and can afford, to pay for access. This should not, and need not, be so.

In the 19th and early 20th centuries independent physicians and small medical societies, interested in making the best new medical knowledge available to doctors, students, and the public, began to publish general medical journals containing case reports, ideas for new treatments, and the results of medical experiments. These pioneers took advantage of the best available technology for disseminating information, printing titles like *The Lancet,* *The New England Journal of Medicine,* and *The Journal of the American Medical Association* on cheap paper and selling them to subscribers at a few pennies a copy. For more than a century, printed journals like these were the dominant means of conveying medical knowledge around the world.

But technology has changed. The Internet is now the most economical and efficient conduit for the delivery of information to most places. Publishers of medical journals realize this—when the Internet took off, they took their journals online. But while they adapted their means of distribution to the 21st century, they left their business model in the 19th century, continuing to charge readers for access just as they had done for their printed journals. This has been good for business—medical publishing has never been more profitable—but it comes at a huge cost. The established medical publishers have turned their back on the opportunity to make the latest and best medical information available to anyone with an Internet connection. With the launch of *PLoS Medicine,* we are embracing this opportunity.

Everything we publish is immediately, freely available online throughout the world, with no restrictions on distribution, copying, printing, or legitimate use.

Everything published in *PLoS Medicine* is immediately freely available online throughout the world, with no restrictions on distribution, copying, printing, or legitimate use. Of course, it costs us money to publish this journal, and we must cover our expenses. But the fee-for-access business model that made perfect sense for the printed journal is no longer consistent with the mission of medical publishing because it needlessly limits the reach of the medical literature. And so we have adopted a new model. Instead of charging readers for access to our journal, we ask the authors of accepted research articles to pay a publication fee to cover the costs of peer review, editorial oversight, and production. This “open access” business model ensures our financial health as a publisher while allowing us to convey everything we publish to the widest possible audience.

Of course, we do not expect authors to cover publication costs personally—rather, we expect the government agencies, companies, foundations, research institutions, hospitals, or universities that sponsor the research to pay the fee. These organizations have always considered the wide dissemination of the results of the research they support to be an integral part of their mission. Virtually every leading sponsor of medical research has announced its willingness to pay for open-access publication, the costs of which average less than one percent of the cost of the research itself—a small price to pay to ensure that everyone who *could* benefit from their research *can* benefit from it.

We realize that not everyone with something important to convey in a medical journal has access to such funds. To ensure that we don't replace a barrier to access with barriers to publication, we've raised money to cover the publication costs of articles whose authors are unable to pay them. And, for every PLoS journal, an author's ability to pay will never be a consideration in our decision to publish an article.

Despite its obvious benefits, open-access publication has met with fierce opposition. Established medical publishers—now businesses more than forces for change—see open-access not as an opportunity to fulfill a mission of public service but as a threat to their lucrative businesses. They contend that their journals still serve the community well, and object that open access threatens their very existence. This is nonsense!

It is our responsibility as publishers and members of the medical community not only to give patients access to the medical literature, but to provide them with tools to use it wisely.

The Wellcome Trust, the world's largest charitable sponsor of biomedical research, seeking to ensure that the results of the science it funds are “disseminated widely and freely available to all,” recently commissioned a thorough analysis of the scientific and medical publishing industry [3]. It concluded that the current market “does not operate in the long-term interest of the research community,” and issued a strong statement in support of open access [4]. Responding to concerns about journal finances, the trust commissioned a detailed economic analysis of open-access publishing [5], based on which it concluded that “the open access model of scientific publishing—where the author of a research paper pays for peer reviewed research to be made available on the web free to all who wish to use it—is economically viable, guarantees high quality research and is a sustainable option which could revolutionise the world of traditional scientific publishing” [6]. (This report, freely available online, is an excellent resource for anyone with questions about the economics of open-access publishing).

We know firsthand that the Wellcome Trust is right. In October 2003, we launched our first journal, *PLoS Biology,* and it is thriving—not only as a destination for the best research in all areas of biology, but also as a resource for students, teachers, and members of the public who have never before had direct access to the product of scientific inquiry (see for yourself at www.plosbiology.org). We are now bringing this success and this spirit to medicine.

The world of medical journals needs a fresh infusion of idealism. All of today's leading medical journals are more than 70 years old, and *PLoS Medicine* is here to challenge the status quo. We are first and foremost an open-access publisher working to ensure that everyone has access to the latest medical research and expertise. But we aim to be more than just an open-access alternative to established general medical journals. We are determined to make *PLoS Medicine* the best medical journal in the world by providing outstanding original research and new ideas; thought-provoking, educational, and imaginative features for readers; and the fastest, fairest, and most rigorous peer review for authors.

As an open-access journal, we see our audience differently than do the conventional medical journals: our audience is composed of medical researchers, physicians, and other health-care providers, patients and their advocates, students, and the public around the world. It will be a great challenge to create a journal that will serve such a diverse audience—we welcome this challenge. We will make it possible for the results of advanced research on infectious diseases to guide treatment in remote clinics thousands of miles away. We will make the results of a clinical trial of a new drug accessible and understandable both to doctors who might prescribe it and to people who might start taking it. We will make research on rare diseases accessible to general practitioners and patients so that they can work together to recognize and treat them.

Whereas some would argue that medical journals should not be accessible to patients because patients are unable to use the information effectively, we believe it is our responsibility as publishers and members of the medical community not only to give patients access, but to provide them with tools to use the medical literature wisely. Medical research is a partnership between medical scientists and millions of voluntary human participants, conducted largely with public funds. What better way to acknowledge the public's contribution and ensure their willingness to sponsor and participate in future research than to openly share the product of this research with them?

We hope that you will enjoy reading *PLoS Medicine* and find it useful and provocative. Please share the journal with your colleagues, patients, and friends. Tell us what you want to see, what you like, and what we could do better. Give us your ideas for changes that will make *PLoS Medicine* a better journal for you and the community. Join us in reinventing the medical journal.
